# Impact of baseline clinical and radiological features on outcome of chronic rhinosinusitis in granulomatosis with polyangiitis

**DOI:** 10.1186/s13075-020-02401-x

**Published:** 2021-01-11

**Authors:** Sigrun Skaar Holme, Karin Kilian, Heidi B. Eggesbø, Jon Magnus Moen, Øyvind Molberg

**Affiliations:** 1 Division of Radiology and Nuclear Medicine, Oslo University Hospital, PB 4950 Nydalen, Oslo, 0424 Norway; 2 Institute of Clincal Medicine, University of Oslo, PB 1072 Blindern, Oslo, 0316 Norway; 3 Department of Rheumatology, Oslo University Hospital, PB 4950 Nydalen, Oslo, 0424 Norway

**Keywords:** Granulomatosis with polyangiitis, Inflammation, Chronic rhinosinusitis, Paranasal sinuses, Osteitis, Saddle nose deformity

## Abstract

**Background:**

Granulomatosis with polyangiitis (GPA) causes a recurring inflammation in nose and paranasal sinuses that clinically resembles chronic rhinosinusitis (CRS) of other aetiologies. While sinonasal inflammation is not among the life-threatening features of GPA, patients report it to have major negative impact on quality of life. A relatively large proportion of GPA patients have severe CRS with extensive damage to nose and sinus structures evident by CT, but risk factors for severe CRS development remain largely unknown. In this study, we aimed to identify clinical and radiological predictors of CRS-related damage in GPA.

**Methods:**

We included GPA patients who had clinical data sets from time of diagnosis, and two or more paranasal sinus CT scans obtained ≥12 months apart available for analysis. We defined time from first to last CT as the study observation period, and evaluated CRS development across this period using CT scores for inflammatory sinus bone thickening (osteitis), bone destructions, and sinus opacifications (here defined as mucosal disease). In logistic regression, we applied osteitis as main outcome measure for CRS-related damage.

**Results:**

We evaluated 697 CT scans obtained over median 5 years observation from 116 GPA patients. We found that 39% (45/116) of the GPA patients remained free from CRS damage across the study observation period, while 33% (38/116) had progressive damage. By end of observation, 32% (37/116) of the GPA patients had developed severe osteitis. We identified mucosal disease at baseline as a predictor for osteitis (odds ratio 1.33), and we found that renal involvement at baseline was less common in patients with severe osteitis at last CT (41%, 15/37) than in patients with no osteitis (60%, 27/45).

**Conclusions:**

In this largely unselected GPA patient cohort, baseline sinus mucosal disease associated with CRS-related damage, as measured by osteitis at the end of follow-up. We found no significant association with clinical factors, but the data set indicated an inverse relationship between renal involvement and severe sinonasal affliction.

**Supplementary Information:**

The online version contains supplementary material available at (10.1186/s13075-020-02401-x).

## Introduction

Granulomatosis with polyangiitis (GPA) is a heterogeneous multisystem disorder causing granulomatous inflammation and necrotising vasculitis in small- and medium-sized vessels. The disease is associated with Anti-Neutrophil Cytoplasmic Antibodies (ANCA), predominantly targeting proteinase 3 (PR3-ANCA). The upper and lower respiratory tract and the kidneys are the organ systems most commonly involved [1–3]. GPA usually runs a relapsing-remitting course with damage accrual. The relapse rates are high, particularly after withdrawal of immunosuppressive therapies [4, 5].

Sinonasal inflammation is common in GPA, with a reported cumulative incidence of at least 80% [4, 6]. Clinical and imaging features indicate an aggressive disease course of the chronic rhinosinusitis (CRS) related to GPA, with pronounced damage to cartilage and bone [7]. Imaging, predominantly paranasal sinus computed tomography (CT) scans, is important for diagnosis and follow-up of CRS in GPA. By CT, sinonasal mucosal thickening and fluid may represent disease activity, while inflammatory bone thickening (defined as osteitis) and destructions presumably represent CRS-related sinus damage.

When GPA patients were asked to rank disease-related challenges in daily life, sinonasal symptoms reached top five together with fatigue, pain, musculoskeletal symptoms, and breathing difficulty [8], explaining why CRS-related features were prioritised items in the newly developed and validated ANCA-associated vasculitis patient-reported outcome measures (AAV-PRO) [9]. Nonetheless, the research on CRS in GPA is still quite limited [6, 10].

Damage accrual, determined by time-dependent increase in the Vasculitis Damage Index (VDI), appears to have major impact on morbidity and mortality in GPA [11]. It is not clear, however if VDI has sufficient resolution to capture extent and development of organ-restricted damage, such as the damage caused by CRS. Follow-up by paranasal sinus CT scans may be a better method to assess sinonasal damage development in GPA. Recently, we developed and applied methods suitable for evaluation of serial paranasal sinus CT scans of GPA patients. We used these methods to assess time-dependent CRS changes, with thickening of the bones of the sinus walls as the main marker for sinus damage [12, 13].

The aim of this study was to identify clinical and radiological baseline predictors of severe CRS outcome, i.e. osteitis, in patients with GPA. Specifically, we analysed the potential impact of clinical variables from time of diagnosis and baseline paranasal sinus CT findings on osteitis. With new knowledge on predictors, we may be able to identify patients at high risk for CRS-related damage already at baseline and provide targeted treatment at early disease stages. Hopefully, this might contribute to reduce the huge negative impact of CRS on GPA patients’ perceived quality of life.

## Methods

### GPA patient cohort and study timeline

**Fig. 1:**
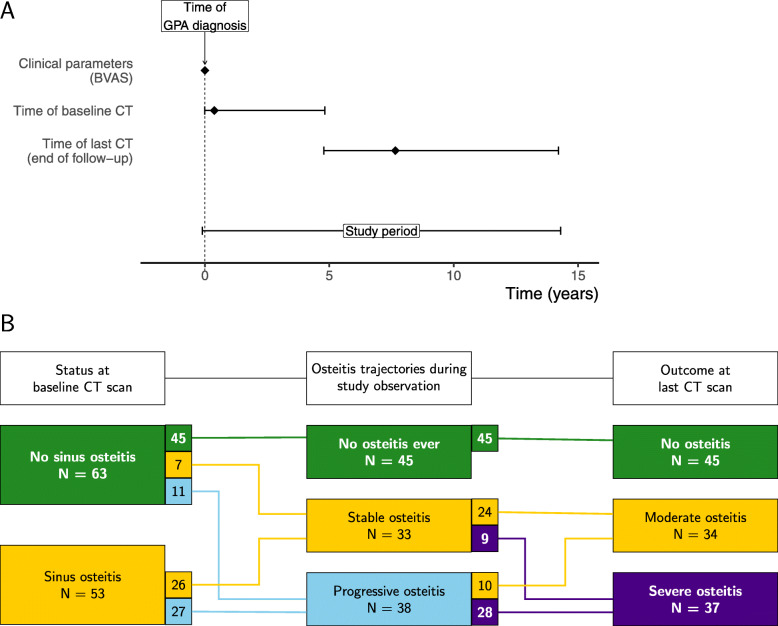
Plot showing the study timeline and osteitis development from baseline to end of follow-up. **a** Timeline plot. Timepoint zero was defined as the year of diagnosis. Median differences between the year of diagnosis and the time of the baseline and the last CT are shown as diamond-shaped points, and the interquartile ranges are shown as horizontal error bars. The mean study observation period with error bars of 2 standard deviations is shown at the bottom. **b** Groups of the study. At baseline, there were two groups. Three different osteitis trajectories ended in three different outcome groups. The connection lines show how the patients changed groups during the study observation period

We have described details on the GPA patient cohort in previous paranasal CT studies [12, 13]. Briefly, the cohort included all the GPA patients from the Norwegian systemic vasculitis and connective tissue disease registry (NOSVAR), a consent-based patient registry run by the Department of Rheumatology at Oslo University Hospital. Pre-specified inclusion criteria were as follows: (I) clinical ANCA vasculitis classifiable as GPA according to the European Medicines Agency (EMA) algorithm [14], and (II) at least two sets of archived sinus CT scans available for analysis, with minimum 1 year between time of baseline CT and time of last CT. Specific to this study, we included patients who had complete electronic medical record (EMR) available from time of GPA diagnosis. We assessed changes in radiological features during the study observation period, defined from the time of baseline CT to the time of last CT in each patient (Fig. 1).

### Clinical parameters assessed at time of GPA diagnosis

From each patient, we reviewed and recorded complete EMR information from time of diagnosis to registration end on 31 December 2016 in a pre-specified medical history form. Form items included the following: date of GPA symptom onset, date of GPA diagnosis assessed by an experienced physician, and type and extent of organ involvement at time of diagnosis defined by the Birmingham Vasculitis Activity Score, version 3 (BVAS) [15]. We also recorded smoking status and the findings on routine blood tests, ANCA tests, and urine analyses. We were able to collect information on medication for the entire observation period for 87 patients. For the remaining 29 patients, only baseline data were available. We defined aggressive first-line treatment as either cyclophosphamide or rituximab treatment or a combination of both. We further chose to stratify the patients according to the definition of the Wegener’s Granulomatosis Etanercept Trial (WGET) research group of limited and severe disease [16], as recommended by EULAR [10] where limited disease equals no organ or life-threatening disease while kidney involvement usually is classified as severe disease.

We retrieved data on paranasal sinus surgery from the EMR and from the Norwegian Patient Registry, which is a mandatory national registry that includes patient identifiable data on surgical procedures from 2008 [13].

### Evaluation and scoring of CRS-related CT findings

We scored CRS-related CT features using scoring systems developed for CRS assessment as previously described [12]. Briefly, we assessed mucosal thickening or fluid (mucosal disease) using the Lund-Mackay score (LM-score) [17], where a completely clear sinus is scored as 0 and a completely opacified sinus as 2, and all degrees of mucosal disease in between are scored as 1. In addition, the ostiomeatal complex is scored as 0 for an open passage and 2 for an obliterated one. For ten sinuses (pairs of frontal, maxillary, and sphenoid sinuses, and pairs of anterior and posterior ethmoid sinuses) and two ostiomeatal complexes, the maximum LM-score is 24.

We assessed bone thickening, i.e. osteitis, using the Global Osteitis Scoring Scale (GOSS) [18], where ten individual sinuses are scored from zero (no osteitis) to 5 (>50*%* of the sinus wall is >5 mm thick), giving a maximum score of 50. We scored bony destruction as present (score 1) or absent (score 0) in 18 defined structures (10 sinuses, 3 pairs of conchae, nasal septum, and hard palate). Saddle nose deformity was evaluated using the lateral contour of the nose seen on the lateral scout view of the CT scans.

We followed the osteitis trajectories of the cohort, and in line with our two previous studies [12, 13], we stratified the patients into three groups based on the osteitis development over time: (I) no osteitis, (II) stable osteitis, and (III) progressive osteitis. Specific to this study, the patients were stratified by osteitis severity at the end of study observation, using a modification of the GOSS severity grading proposed by Georgalas et al. [18]. Three groups were defined: (I) no osteitis (GOSS = 0), (II) mild to moderate osteitis with GOSS <35 and no single sinus with score >4, and (III) severe osteitis with GOSS ≥35 or GOSS <35 and at least one sinus score = 5 (Fig. 1).

### Statistical analyses

Normally distributed variables were given in mean (SD) and skewed variables in median (range) where range is from the minimum value to the maximum value. Proportions were given in number (percentage). Mean difference with 95% confidence interval was calculated by t-distribution. Due to skewed data, we calculated correlation by Spearman’s rank correlation coefficient with a bias-corrected and accelerated (BCa) bootstrap 95% confidence interval.

Logistic regression modelling was used to assess the strength of association between selected covariates and osteitis at last CT, which was our CRS-related outcome variable. Some of the patients had undergone sinus surgery either before or during observation. We excluded patients after they had undergone surgery in the present modelling data set, to avoid time-varying covariates. Patients who had at least 1 year of observation before their first surgery were included with their preoperative observation time (*n* = 102). Covariates were baseline mucosal thickening or fluid (mucosal disease) scored by LM-score, renal involvement at baseline by BVAS, age at diagnosis, sex, disease duration at the baseline CT, and the time between baseline CT and last CT (i.e. observation period). Medication was not included in the model as it is a time-varying covariate. Level of significance was set at <0.05.

## Results

### GPA patient cohort, demographics, and clinical characteristics at time of diagnosis

The GPA cohort consisted of 116 patients. Mean age at diagnosis was 45 (SD = 19) years and 54% were men. ANCA was positive in 109 patients. Sixty-four patients (55%) had severe GPA as defined by the WGET research group criteria [16] (Table 1). At the time of GPA diagnosis, 37/116 (32%) patients had concurrent involvement of upper and lower airways and kidneys, while 26/116 (22%) had combined upper and lower airway diseases, with no renal involvement. Symptoms and/or signs of upper airway disease were present in 107/116 (92%) of the GPA patients, with involvement of the nose and/or sinuses in 102 of them. Sinus involvement at baseline was seen more often in men (76%, 48/63) than in women (58%, 31/53), and more men (63%; 40/63) than women (42%; 22/53) had baseline kidney involvement.

**Table 1:** Demographics, baseline clinical data, and baseline CT scores in total cohort

	Total,	No baseline osteitis,	Baseline osteitis,
	*n* = 116	*n* = 63	*n* = 53
**Demographic data**			
Women; *n* (%)	53 (46)	32 (51)	21 (40)
Limited GPA, as defined in WGET research group; *n* (%)	52 (45)	26 (41)	26 (49)
Mean age at diagnosis in years (SD)	45 (19)	45 (20)	46 (19)
Median time in years between symptom onset and diagnosis (range)	0.5 (0.0–10.0)	0.5 (0.0–9.3)	0.5 (0.0–10.0)
	Missing = 1	Missing = 1	Missing = 0
Median time in years from diagnosis to first CT (range)	0.3 (−3.9–24.6)	0.0 (−3.9–18.5)	0.8 (−1.3–24.6)
Ever smokers; fraction (%)	48/94 (51)	26/51 (51)	22/43 (51)
Mortality; *n* (%)	10 (9)	4 (6)	6 (11)
**Laboratory data**			
ANCA positive; fraction (%)	109/110 (99)	62/63 (98)	47/47 (100)
ANCA negative; fraction (%)	1/110 (0.9)	1/63 (1.6)	0/47 (0)
**Clinical data, BVAS**			
General; *n* (%)	107 (92)	56 (89)	51 (96)
Cutaneous; *n* (%)	26 (22)	15 (24)	11 (21)
Mucosal membranes/eyes; *n* (%)	33 (28)	19 (30)	14 (26)
Ear, nose, and throat; *n* (%)	107 (92)	59 (94)	48 (91)
Bloody nasal discharge/crusts/ulcers/granulomata; *n* (%)	93 (80)	50 (79)	43 (81)
Paranasal sinus involvement; *n* (%)	79 (68)	37 (59)	42 (79)
Subglottic stenosis; *n* (%)	0 (0)	0 (0)	0 (0)
Conductive hearing loss; *n* (%)	33 (28)	23 (37)	10 (19)
Sensorineural hearing loss; *n* (%)	7 (6)	7 (11)	0 (0)
Chest; *n* (%)	69 (59)	36 (57)	33 (62)
Cardiovascular; *n* (%)	1 (1)	1 (2)	0 (0)
Abdominal; *n* (%)	6 (5)	4 (6)	2 (4)
Renal; *n* (%)	62 (53)	34 (54)	28 (53)
Nervous system; *n* (%)	21 (18)	13 (21)	8 (15)
**Surgery before baseline**			
Sinus surgery; *n* (%)	12 (10)	1 (2)	11 (21)
**CT scores**			
Median GOSS (range)	0 (0–38)	0 (0–0)	4 (1–38)
Sinonasal destructions; *n* (%)	29 (25)	5 (8)	24 (45)
Median LM-score (range)	3 (0–22)	2 (0–13)	6 (0–22)
LM-score >0; *n* (%)	95 (82)	44 (70)	51 (96)

Missing data on ANCA and smoking

*Abbreviations:GPA* granulomatosis with polyangiitis, *WGET* Wegener’s Granulomatosis Etanercept Trial, *ANCA* anti-neutrophil cytoplasmic antibodies, *BVAS* Birmingham Vasculitis Activity Score, version 3, *GOSS* Global Osteitis Scoring Scale, *LM-score* Lund-Mackay score

### Changes in paranasal sinus CT findings from baseline to end of follow-up

We evaluated 697 sinus CT scans from the 116 patients, with median 5 CT scans per patient. From 68/116 patients, we had baseline CT scans performed within 1 year from the time of GPA diagnosis (Fig. 1).

At the baseline CT scan, we found that of the 116 patients, 95 (82%) had mucosal thickening or fluid in the sinuses (defined by LM-score >0), 29 (25%) had bony destructions (Table 1), and 53 (46%) had osteitis (Fig. 1). The patients who had osteitis at baseline had a longer delay between year of diagnosis and year of baseline CT (mean difference = 2.8; 95% confidence interval 0.7 to 4.9).

To determine changes in CRS-related damage during the median 5-year study observation period, we scored osteitis severity (GOSS) on all CT scans performed during follow-up. In line with previously published data on this cohort [12], we found that the degree of osteitis either remained stable or progressed during observation. In this cohort, 53 patients had osteitis at the baseline CT, 26 of these had stable osteitis during observation, while 27 had progressive osteitis (Fig. 1). Time-course analyses of the 63 patients with no signs of osteitis at the baseline CT showed that 45 patients remained free from osteitis during observation, seven developed a mild osteitis that remained stable across the observation period, and 11 displayed progressive osteitis (Fig. 1).

At the end of the study period, defined for each patient as the time of the last sinus CT scan, outcomes regarding CRS-related damage were as follows: 45 patients had no osteitis, 34 patients had moderate osteitis, and 37 had developed severe osteitis (Fig. 1).

Analyses of additional CT damage parameters showed that 15/116 (13%) had nasal septum perforation (*n*=12) and/or saddle nose deformity (*n*=8) already at baseline. At the end of follow-up, 30/116 (26%) patients had saddle nose and/or nasal septum perforation, with saddle nose in 16 and nasal septum perforation in 26. Sixty percent (18/30) of the patients who had saddle nose and/or nasal septum perforation at end of observation had at the time of diagnosis limited disease, according to the definition of WGET.

### Associations between baseline radiological features and outcome of CRS

**Fig. 2:**
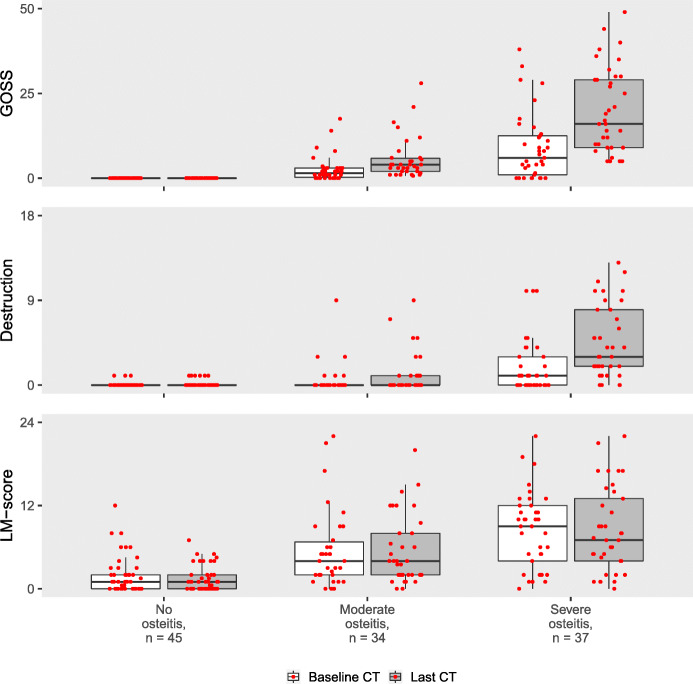
The levels of the three CT parameters at baseline and last CT. Upper panel shows bone thickening (i.e. osteitis) measured by Global Osteitis Scoring Scale (GOSS), number of destructed sinonasal bone structures in the middle panel, and sinonasal mucosal disease measured by the Lund-Mackay score (LM-score) in the bottom panel. The variables are grouped by the degree of osteitis severity at last CT. The observations are shown as red dots

As expected, we found the most pronounced increase in osteitis from baseline to end of study in the patient subset with a severe osteitis outcome (Fig. 2). Regarding mucosal disease, we found gradually increasing median baseline LM-score from the no osteitis group via the moderate to the severe osteitis group (Table 2 and Fig. 2). Consistently, the baseline LM-score correlated with GOSS at last CT (*ρ*=0.64 with 95% confidence interval 0.50 to 0.75).

**Table 2:** Demographics, baseline clinical data, and CT scores at baseline and last CT grouped by osteitis severity at last CT

	No osteitis,	Moderate osteitis,	Severe osteitis,
	*n* = 45	*n* = 34	*n* = 37
**Demographic data**			
Women; *n* (%)	23 (51)	15 (44)	15 (41)
Limited GPA, as defined in WGET research group; *n* (%)	18 (40)	15 (44)	19 (51)
Mean age at diagnosis in years (SD)	44 (20)	54 (18)	39 (18)
Median time in years between symptom onset and diagnosis (range)	44; 0.4 (0.0–9.3)	0.7 (0.0–6.0)	0.4 (0.1–10.0)
	Missing = 1		
Median time in years from diagnosis to first CT (range)	0.1 (−3.9–18.5)	0.3 (−2.3–16.1)	0.5 (−1.3–24.6)
Median time in years between first and last CT (range)	4.5 (1.3–12.9)	4.8 (1.1–13.5)	7.3 (2.1–14.7)
Ever smokers; fraction (%)	17/36 (47)	14/30 (47)	17/28 (61)
Mortality; *n* (%)	3 (7)	3 (9)	4 (11)
**Laboratory data**			
ANCA positive; fraction (%)	44/45 (98)	33/33 (100)	32/32 (100)
ANCA negative; fraction (%)	1/45 (2.2)	0/33 (0)	0/32 (0)
**Baseline clinical data, BVAS**			
General; *n* (%)	42 (93)	32 (94)	33 (89)
Cutaneous; *n* (%)	11 (24)	7 (21)	8 (22)
Mucosal membranes/eyes; *n* (%)	14 (31)	10 (29)	9 (24)
Ear, nose, and throat; *n* (%)	42 (93)	29 (85)	36 (97)
Bloody nasal discharge/crusts/ulcers/granulomata; *n* (%)	35 (78)	25 (74)	33 (89)
Paranasal sinus involvement; *n* (%)	23 (51)	23 (68)	33 (89)
Subglottic stenosis; *n* (%)	0 (0)	0 (0)	0 (0)
Conductive hearing loss; *n* (%)	15 (33)	6 (18)	12 (32)
Sensorineural hearing loss; *n* (%)	5 (11)	1 (3)	1 (3)
Chest; *n* (%)	26 (58)	21 (62)	22 (59)
Cardiovascular; *n* (%)	1 (2)	0 (0)	0 (0)
Abdominal; *n* (%)	3 (7)	1 (3)	2 (5)
Renal; *n* (%)	27 (60)	20 (59)	15 (41)
Nervous system; *n* (%)	7 (16)	4 (12)	10 (27)
**Surgery at end of follow-up**			
Sinus surgery; *n* (%)	1 (2)	4 (12)	17 (46)
**CT scores at baseline**			
Median GOSS (range)	0 (0–0)	2 (0–18)	6 (0–38)
Sinonasal destructions; *n* (%)	3 (7)	6 (18)	20 (54)
Nasal septum perforation; *n* (%)	3 (7)	4 (12)	5 (14)
Saddle nose deformity; *n* (%)	2 (4)	1 (3)	5 (14)
Median LM-score (range)	1 (0–12)	4 (0–22)	9 (0–22)
LM-score >0; *n* (%)	29 (64)	30 (88)	36 (97)
**CT scores at last CT**			
Median GOSS (range)	0 (0–0)	4 (1–28)	16 (5–49)
Sinonasal destructions; *n* (%)	6 (13)	11 (32)	33 (89)
Nasal septum perforation; *n* (%)	6 (13)	7 (21)	13 (35)
Saddle nose deformity; *n* (%)	2 (4)	4 (12)	10 (27)
Median LM-score (range)	1 (0–7)	4 (0–20)	7 (0–22)

Missing data on ANCA and smoking

*Abbreviations:GPA* granulomatosis with polyangiitis, *WGET* Wegener’s Granulomatosis Etanercept Trial, *ANCA* anti-neutrophil cytoplasmic antibodies, *BVAS* Birmingham Vasculitis Activity Score, version 3, *GOSS* Global Osteitis Scoring Scale, *LM-score* Lund-Mackay score

Additional file 1: Figure S1 shows the relationship between longitudinal measurements of LM-score compared to GOSS in GPA cohort patients with no history of paranasal sinus surgery, available clinical data on medical treatment, and a timespan from baseline clinical data to first CT of <2 years (*n*=68). The figure indicates distribution of baseline LM-score in the patients stratified by osteitis trajectories. We identified increasing osteitis following a CT scan with a high LM-score in individual patients. Additional file 2: Figure S2 shows the same patients grouped by three levels of baseline LM-score, with timelines for osteitis development and treatment with rituximab/cyclophosphamide showed for individual patients. We were not able to show any clear effects of the treatment on the osteitis development.

Finally, the patients with severe osteitis outcome also had more bony destruction including saddle nose deformity than the no osteitis and moderate osteitis outcome groups (Fig. 2, Table 2), but it should be noted that surgical interventions in sinuses, which often include removal of bony structures, were more prevalent in the severe osteitis group (Table 2).

### Associations between baseline demographics, clinical characteristics, and outcome of CRS

**Fig. 3:**
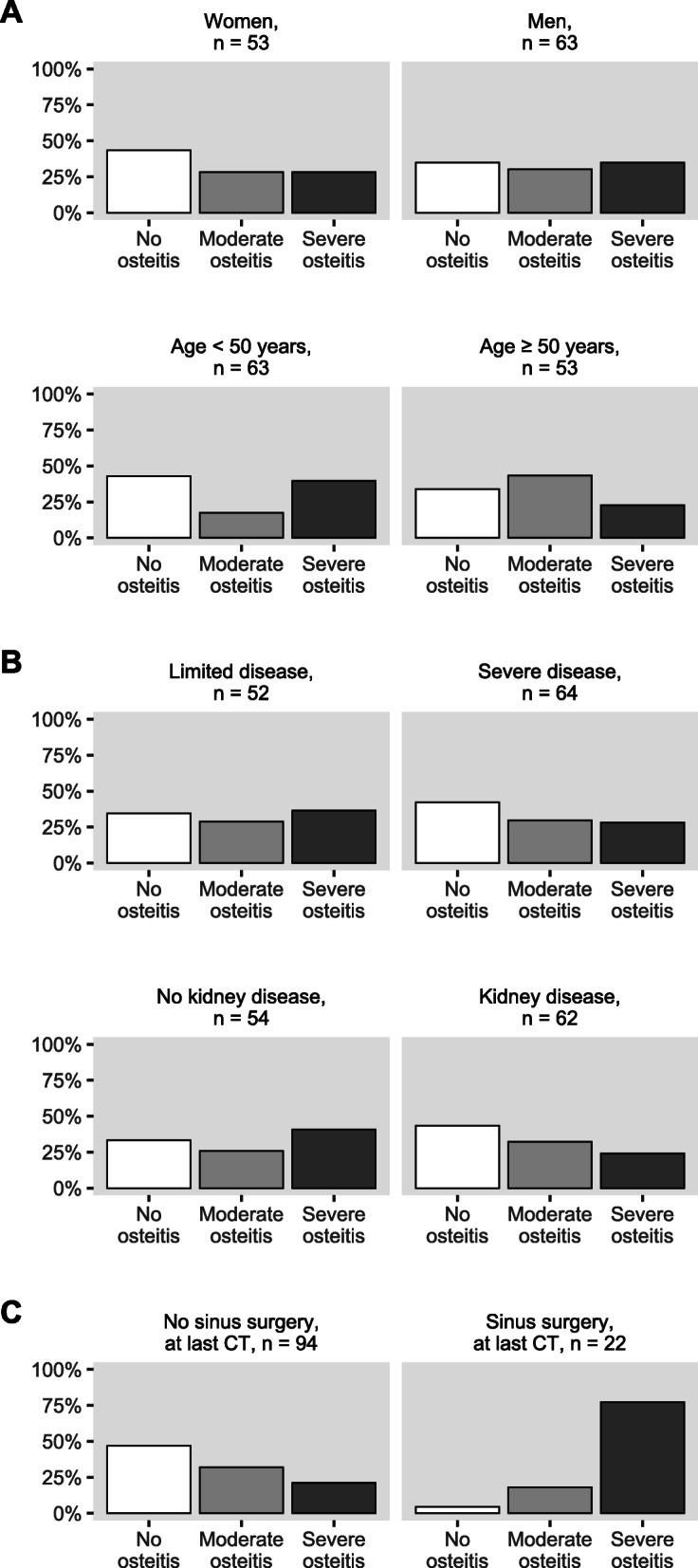
Associations to the degree of osteitis severity at last CT. Demographic data including age at diagnosis (**a**), baseline clinical data (**b**), and total sinus surgery (**c**). Limited/severe disease is by the definition of the WGET research group

When patients were stratified by outcome of CRS, we found that the group ending with severe osteitis (*n*=37) were younger at disease onset than the patients with no osteitis (*n*=45) or moderate osteitis (*n*=34) and included more smokers (Table 2). In patients who were <50 years at disease onset, we found that 40% (25/63) had a severe osteitis outcome, compared to 23% (12/53) in patients with onset >50 years (Fig. 3). Severe osteitis outcome was slightly more frequent in men (35%, 22/63) than in women (28%, 15/53). Overall, we found that types and extent of organ involvement at baseline were quite similar across the osteitis outcome groups (Table 2). However, in patients with renal involvement at baseline (as defined by BVAS), severe osteitis outcome was less frequent (24%, 15/62) than in patients with no renal disease (41%, 22/54). We found a similar difference with disease classified using WGET, where 28% (18/64) with severe disease and 37% (19/52) with limited disease developed severe osteitis (Fig. 3).

We had complete data sets on the aggressive first-line therapies (cyclophosphamide or rituximab) given during observation in 87 GPA cohort patients. In these 87 patients, we found that 100% (44/44) of those with baseline kidney disease and 79% (34/43) with no kidney disease at baseline received aggressive first-line treatment. Thus, only nine patients never had any treatment with cyclophosphamide or rituximab. Of these nine patients, five had no osteitis at last CT and four had moderate osteitis. The aggressive first-line treatment group of patients (*n*=78) were distributed between all three osteitis severity groups: 41% (32/78) had no osteitis at last CT, 33% (26/78) had moderate osteitis, and 26% (20/78) had severe osteitis.

Surgery is associated to osteitis [13, 20]. Consistently, we found a large difference in osteitis outcome between GPA patients with or without a history of sinus surgery (Fig. 3). Therefore, we removed the surgery patients (7/87) from the group of patients having complete data on medication. Osteitis development and timing of cyclophosphamide or rituximab medication are shown in these patients in Additional file 2: Figure S2. After removing the surgery patients, a lesser proportion of the patients had severe osteitis (13/80, 16%). We selected the patients who had aggressive first-line treatment (71/80) and stratified them on kidney disease and found that a higher proportion of patients without baseline kidney disease had severe osteitis at last CT (29%, 8/28) than of patients with baseline kidney disease (12%, 5/43).

### Baseline clinical and radiological features as risk factors for osteitis

Patients were excluded after sinus surgery from the modelling, as detailed in the “Methods” section. We excluded surgery and medical treatment as covariates in the model since they were time-varying variables.

A logistic regression model was fitted with baseline covariates and the outcome variable which divided the patients into two groups (no osteitis and a merged moderate and severe osteitis group). We found a clear association between baseline LM-score and the osteitis outcome variable, where the odds of having osteitis at last CT increased by 33% for one-unit increase in baseline LM-score with 95% confidence interval from 17% increase in odds to 57%. Baseline renal disease and sex were included in multivariable analyses but were not significant (Table 3).

**Table 3:** Univariable and multivariable logistic regression models with outcome variable osteitis at last CT. Estimates (odds ratio), 95% confidence intervals (CI), and *p* values

	Univariable	95% CI		*p* value	Multivariable	95% CI		*p* value
(Intercept)	–	–	–	–	0.62	0.27	1.39	0.25
LM-score	1.33	1.17	1.57	7.5e −05	1.35	1.18	1.6	1.13e −04
Kidney disease	0.59	0.26	1.29	0.19	0.71	0.28	1.79	0.46
Men	1.25	0.57	2.74	0.58	0.73	0.27	1.89	0.52
Age at diagnosis	1.01	0.99	1.03	0.24				
Disease duration at baseline (years)	1.02	0.95	1.1	0.6				
Observation period (years)	1.02	0.91	1.16	0.7				

*Abbreviation:LM-score* Lund-Mackay score

## Discussion

CRS is highly prevalent in GPA and a major contributor to perceived disease burden. Still, we know little about risk factors for severe CRS outcomes in GPA. In this study, the main finding was that baseline sinonasal CT findings consistent with mucosal disease increased the risk for development and progression of CRS-related damage (osteitis) during follow-up. We found no effect of age at diagnosis or disease duration at the time of the baseline CT (OR = 1), but the results indicated, albeit not statistically significant, that baseline kidney involvement reduced the odds.

Our finding of a positive correlation between baseline mucosal disease measured by LM-score and development of osteitis measured by GOSS in GPA is novel. A previous cross-sectional study described associations between LM-score and osteitis in CRS [18], but this was not investigated in studies on GPA-related CRS [7].

In this study, we used sinonasal osteitis rather than destructions as primary marker of CRS-related damage, because we had previously observed in individual patients that it was highly challenging to differentiate bony destructions caused by CRS from post-surgical bone changes [13]. Since no prospective studies exist on CRS in GPA, we do not know if CRS-related damage correlates with sinonasal symptom severity. However, we do know that sinonasal complaints and sinonasal osteitis both are common in GPA patients [6, 8]. In CRS, a prospective follow-up study showed that patients with severe osteitis at baseline experienced less symptom improvement after the surgical intervention than patients without osteitis [19].

In addition to osteitis, we focused on saddle nose deformity and nasal septum perforation as clinically important organ damage. Few GPA studies have assessed these nose complications by paranasal CT scans [7], and only one of these studies included more than 15 patients [20]. This cross-sectional study from a tertiary otolaryngology centre by Grindler et al. reported CT findings consistent with saddle nose in 34% (25/74) of GPA patients and septal perforation in 35% [20]. For comparison, by end of follow-up, we identified septal perforation and saddle nose in 22% (26/116) and 14% (16/116) of patients, respectively. This difference indicates probably selection for GPA patients with severe sinonasal disease in Grindler’s cohort.

Although no variables other than baseline mucosal disease were significant in the logistic regression model, some clinical variables did show a different distribution between the osteitis groups. Specifically, patients with no renal involvement and/or limited GPA (by the WGET definition) at baseline seemed to have increased risk for severe osteitis development. The potential negative association between baseline kidney involvement and severe CRS is, to our knowledge, a new observation. It is, however, indirectly supported by the finding of low frequency of renal disease (27%) in the abovementioned GPA cohort from Grindler et al. [20], where a high proportion of patients had severe CRS with osteitis (78%).

The positive correlation between baseline kidney disease and severe osteitis might be due to more aggressive systemic treatment of the patients with kidney disease. In our cohort, 90% (78/87) of the patients with complete data on medical treatment had cyclophosphamide and/or rituximab treatment. This is probably the major reason why we were not able to show any differences in osteitis development related to treatment. The different frequency of severe osteitis between patients with or without baseline kidney disease remained when comparing the cyclophosphamide/rituximab-treated patients. The amount of cyclophosphamide or rituximab were not accounted for.

Finally, it seemed that younger age at diagnosis (<50 years as compared to ≥50 years) associated with development of severe osteitis during observation (Fig. 3). The difference was perhaps due to longer disease duration in the younger patients with mean increased disease duration of 4.8 years with 95% confidence interval 2.2 to 7.3 years. However, twice as many younger patients had undergone sinus surgery which could reflect a more aggressive disease in the younger and partly explain the higher level of osteitis. In the logistic regression model, where the surgery patients were excluded, the age at diagnosis had no effect (odds ratio = 1). The potential effect of sex on osteitis was unclear, with trends in opposite directions in the uni- and multivariable analyses. We do, however, believe that the relatively even distributions of males across the three categories of osteitis at study end (as shown in Fig. 3) argue against sex differences regarding severe CRS damage.

### Limitations and strengths

The major strength of this study is that it contains detailed analyses of a large number of paranasal sinus CT scans obtained over a relatively long observation period from a largely unselected cohort of GPA patients. Due to the unselected nature of the cohort, we were able to identify groups of patients with different CRS trajectories, ranging from patients with no osteitis ever to patients with progressive changes and severe osteitis outcome.

An obvious weakness, which relates to the observational study design, is that we did not have baseline CT from time of GPA diagnosis in all the patients. Additionally, we could not develop a prediction model for osteitis because of too little data, and we were not able to fit any model with more than one significant variable.

Furthermore, the study did not take into account the underlying systemic activity of GPA at time of CT scanning. As previously mentioned, BVAS is the recommended measure of disease activity in GPA. We have retrospectively scored BVAS at baseline in this study, but did not have data on BVAS from the time of each CT scan. However, the efficacy of BVAS as a disease activity measure for the sinonasal area in GPA is under debate since BVAS has been considered too coarse. Decker et al. [21] proposed therefore an endoscopy-based disease activity score in GPA, but this required follow-up by otorhinolaryngologists.

We think that our clear association between baseline mucosal disease and osteitis at last follow-up could suggest LM-score as a potential useful indicator of activity. Our longitudinal data on mucosal disease measured by LM-score and osteitis also suggests that LM-score measured at each follow-up CT might be useful as a disease activity measure. This needs confirmation in independent GPA populations in prospective studies with longitudinal data and preferably comparison to both endoscopy findings and BVAS.

### Conclusion

We found a clear association between baseline paranasal sinus mucosal disease and osteitis at last follow-up (odds ratio = 1.33 with 95% confidence interval 1.17 to 1.57). No other variables reached the significance level of 5%, but there seemed to be a negative association between osteitis and kidney disease even in patients who had cyclophosphamide or rituximab treatment, indicating that development of severe CRS damage is not evenly distributed across the GPA population.

## Supplementary Information

**Additional file 1** Longitudinal relationship between sinus mucosal disease measured by Lund-Mackay score (LM-score) and osteitis measured by Global Osteitis Scoring Scale (GOSS) in selected patients of the three osteitis trajectory groups. (A) Distribution of baseline LM-score. (B) Subplots showing GOSS and LM-scores for each CT in each of the selected patients of the three osteitis trajectory groups. A vertical coloured line indicates the start of treatment with cyclophosphamide or rituximab in each patient.

**Additional file 2** Influence of baseline sinus mucosal disease, baseline kidney disease and treatment with rituximab or cyclophosphamide on osteitis development. (A) Distribution of osteitis measured by Global osteitis scoring scale (GOSS) in three groups of patients defined by baseline Lund-Mackay score (LM-score). (B) Subplots showing GOSS for each CT and a vertical coloured line for start of treatment with cyclophosphamide or rituximab in each patient. The patients had follow-up data on medication and had a baseline CT less than two years after the year of diagnosis.

## Data Availability

The data sets used during the current study are available from the corresponding author on reasonable request.

## References

[1] Jennette JC, Falk RJ, Andrassy K, Bacon PA, Churg J, Gross WL (1994). Nomenclature of systemic vasculitides. Arthritis Rheum.

[2] Jennette JC, Falk RJ, Bacon PA, Basu N, Cid MC, Ferrario F (2013). 2012 Revised international chapel hill consensus conference nomenclature of vasculitides. Arthritis Rheum.

[3] Leavitt RY, Fauci AS, Bloch DA, Michel BA, Hunder GG, Arend WP (1990). The American College of Rheumatology 1990 criteria for the classification of Wegener’s granulomatosis. Arthritis Rheum.

[4] Holle JU, Gross WL, Latza U, NÖlle B, Ambrosch P, Heller M, Fertmann R, Reinhold–Keller E (2011). Improved outcome in 445 patients with Wegener’s granulomatosis in a German vasculitis center over four decades. Arthritis & Rheum.

[5] Puéchal X, Pagnoux C, Hamidou M, Boffa JJ, Kyndt X, Lifermann F, Papo T, Merrien D, Smail A, Delaval P, Perrodeau É (2016). Long-term outcomes among participants in the WEGENT trial of remission-maintenance therapy for granulomatosis with polyangiitis (Wegener’s) or microscopic polyangiitis. Arthritis Rheumatol.

[6] Kühn D, Hospowsky C, Both M, Hey M, Laudien M (2018). Manifestation of granulomatosis with polyangiitis in head and neck. Clin Exp Rheumatol.

[7] D’Anza B, Langford CA, Sindwani R (2017). Sinonasal imaging findings in granulomatosis with polyangiitis (Wegener granulomatosis): a systematic review. Am J Rhinol Allergy.

[8] Herlyn K, Hellmich B, Seo P, Merkel PA (2010). Patient-reported outcome assessment in vasculitis may provide important data and a unique perspective. Arthritis Care Res.

[9] Robson JC, Dawson J, Doll H, Cronholm PF, Milman N, Kellom K (2018). Validation of the ANCA-associated vasculitis patient-reported outcomes (AAV-PRO) questionnaire. Ann Rheum Dis.

[10] Hellmich B, Flossmann O, Gross WL, Bacon P, Cohen-Tervaert JW, Guillevin L (2007). EULAR recommendations for conducting clinical studies and/or clinical trials in systemic vasculitis: focus on anti-neutrophil cytoplasm antibody-associated vasculitis. Ann Rheum Dis.

[11] Exley A, Bacon P, Luqmani R, Kitas G, Gordon C, Savage C (1997). Development and initial validation of the vasculitis damage index for the standardized clinical assessment of damage in the systemic vasculitides. Arthritis Rheum.

[12] Holme SS, Moen JM, Kilian K, Haukeland H, Molberg Ø, Eggesbø HB (2019). Development of CT-based methods for longitudinal analyses of paranasal sinus osteitis in granulomatosis with polyangiitis. BMC Med Imaging.

[13] Holme SS, Moen JM, Kilian K, Eggesbø HB, Molberg Ø (2020). Impact of paranasal sinus surgery in granulomatosis with polyangiitis: a longitudinal computed tomography study. Laryngoscope.

[14] Watts R, Lane S, Hanslik T, Hauser T, Hellmich B, Koldingsnes W (2007). Development and validation of a consensus methodology for the classification of the ANCA-associated vasculitides and polyarteritis nodosa for epidemiological studies. Ann Rheum Dis.

[15] Mukhtyar C, Lee R, Brown D, Carruthers D, Dasgupta B, Dubey S (2009). Modification and validation of the Birmingham Vasculitis Activity Score (version 3). Ann Rheum Dis.

[16] The WGET Research Group. Design of the Wegener’s granulomatosis etanercept trial (WGET). Control Clin Trials. 2002; 23(4):450–68. 10.1016/s0197-2456(02)00209-x 12161090

[17] Lund VJ, Mackay IS (1993). Staging in rhinosinusitus. Rhinology.

[18] Georgalas C, Videler W, Freling N, Fokkens W (2010). Global Osteitis Scoring Scale and chronic rhinosinusitis: a marker of revision surgery. Clin Otolaryngol.

[19] Bhandarkar ND, Mace JC, Smith TL (2011). The impact of osteitis on disease severity measures and quality of life outcomes in chronic rhinosinusitis. Int Forum Allergy Rhinol.

[20] Grindler D, Cannady S, Batra PS (2009). Computed tomography findings in sinonasal Wegener’s granulomatosis. Am J Rhinol Allergy.

[21] Decker L, Türp L, Borzikowsky C, Laudien M (2017). Intra-and inter-rater reliability of the modified ENT assessment score (ENTAS 2) in granulomatosis with polyangiitis: a prospective randomised trial. Clin Exp Rheumatol.

