# Supplementary figures and images for: Impact of baseline clinical and radiological features on outcome of chronic rhinosinusitis in granulomatosis with polyangiitis
Source: Arthritis Res Ther. 2021 Jan 11;23:18. doi: 10.1186/s13075-020-02401-x (PMC7802308; doi:10.1186/s13075-020-02401-x)

**A**

No osteitis, n = 31

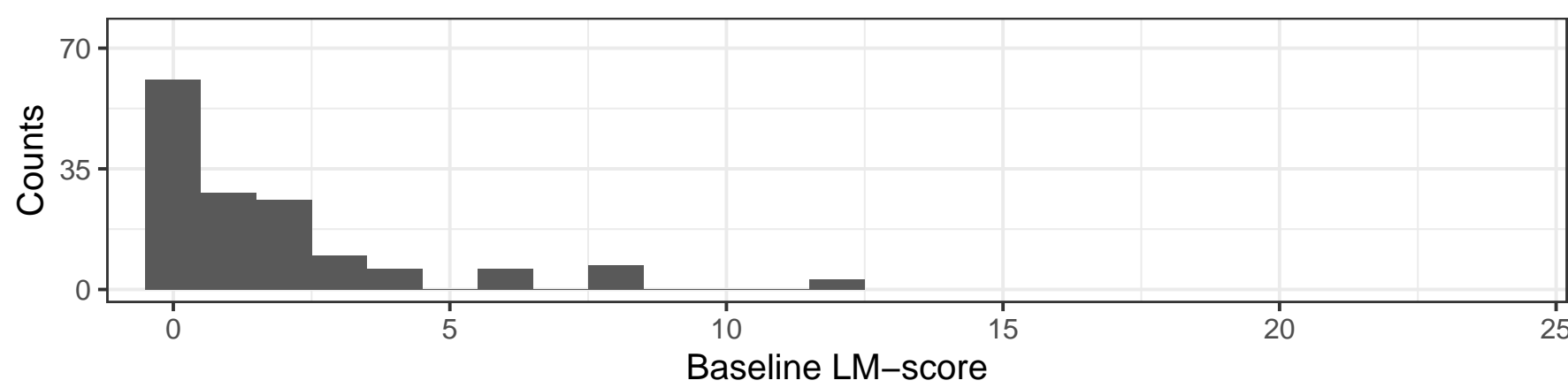

Stable osteitis, n = 15

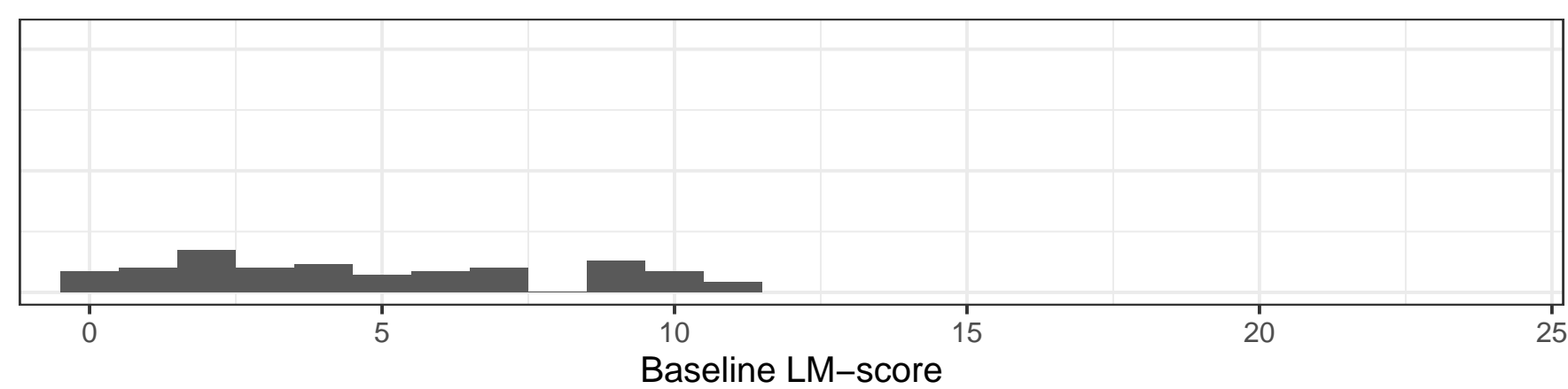

Progressive osteitis, n = 22

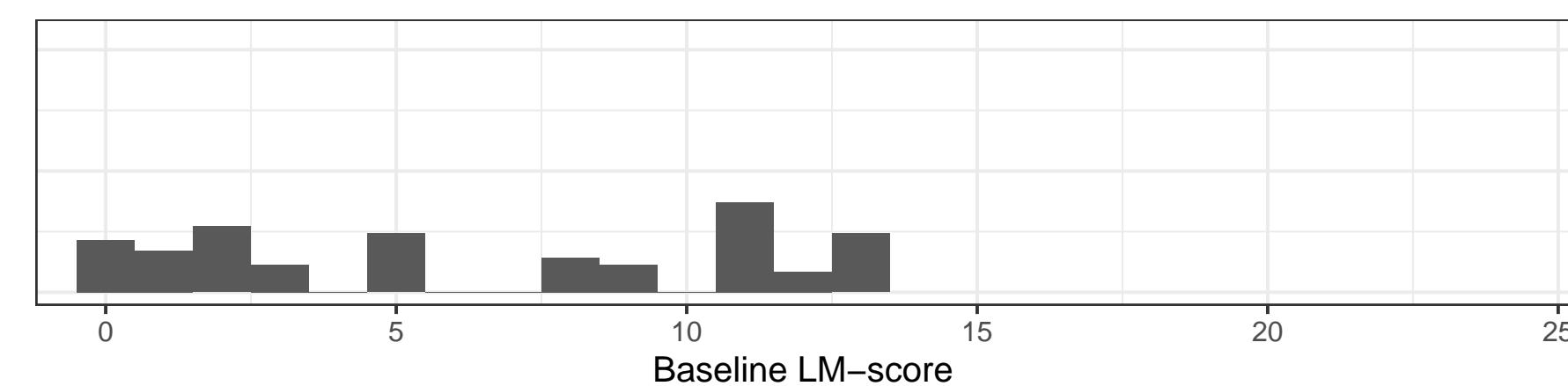**B**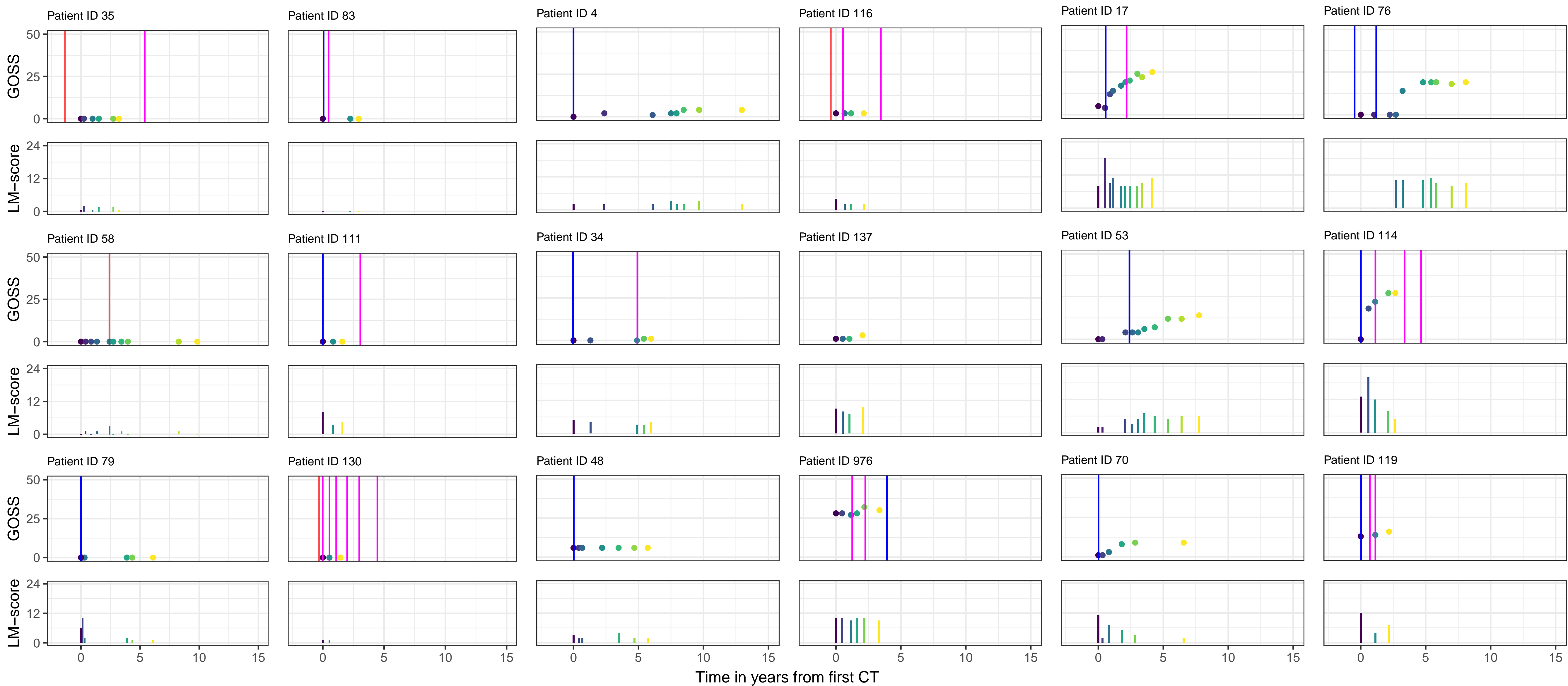

Both Cyclophosphamide Rituximab

Supplement: Supplementary file 1 — Additional file 1 Longitudinal relationship between sinus mucosal disease measured by Lund-Mackay score (LM-score) and osteitis measured by Global Osteitis Scoring Scale (GOSS) in selected patients of the three osteitis trajectory groups. (A) Distribution of baseline LM-score. (B) Subplots showing GOSS and LM-scores for each CT in each of the selected patients of the three osteitis trajectory groups. A vertical coloured line indicates the start of treatment with cyclophosphamide or rituximab in each patient. [file 13075_2020_2401_MOESM1_ESM.pdf]

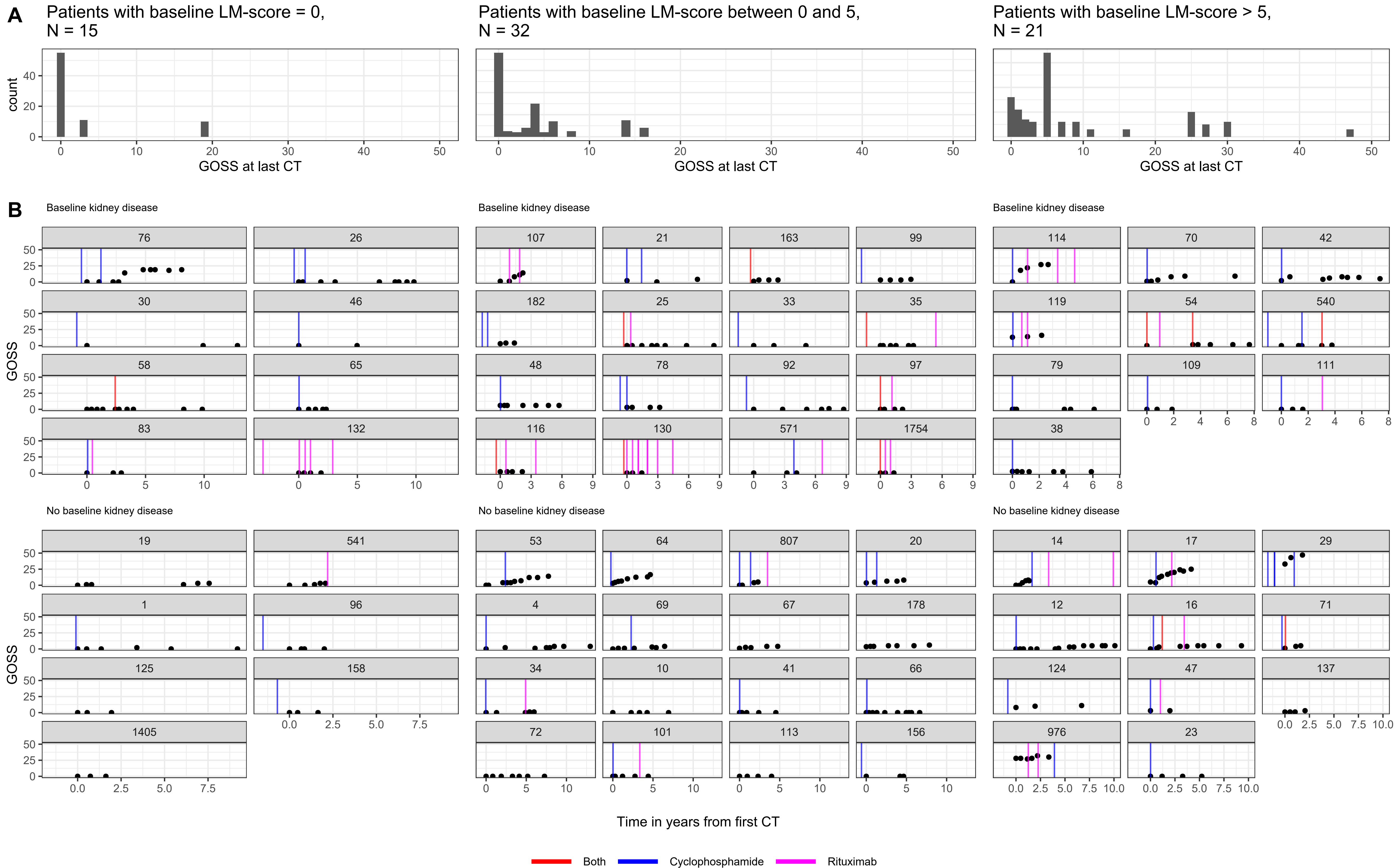

Supplement: Supplementary file 2 — Additional file 2 Influence of baseline sinus mucosal disease, baseline kidney disease and treatment with rituximab or cyclophosphamide on osteitis development. (A) Distribution of osteitis measured by Global osteitis scoring scale (GOSS) in three groups of patients defined by baseline Lund-Mackay score (LM-score). (B) Subplots showing GOSS for each CT and a vertical coloured line for start of treatment with cyclophosphamide or rituximab in each patient. The patients had follow-up data on medication and had a baseline CT less than two years after the year of diagnosis. [file 13075_2020_2401_MOESM2_ESM.pdf]
